# Optimizing the Synthetic Conditions of “Green” Colloidal AgBiS_2_ Nanocrystals Using a Low-Cost Sulfur Source

**DOI:** 10.3390/nano12213742

**Published:** 2022-10-25

**Authors:** Qiao Li, Xiaosong Zheng, Xiaoyu Shen, Shuai Ding, Hongjian Feng, Guohua Wu, Yaohong Zhang

**Affiliations:** 1 School of Physics, Northwest University, Xi’an 710127, China; 2 Qingdao Innovation and Development Base of Harbin Engineering University, Harbin Engineering University, Harbin 150001, China; 3 Shaanxi Key Laboratory for Carbon Neutral Technology, Xi’an 710127, China

**Keywords:** AgBiS_2_, colloidal nanocrystals, hot injection, sulfur source

## Abstract

Colloidal AgBiS_2_ nanocrystals (NCs) have attracted increasing attention as a near–infrared absorbent materials with non–toxic elements and a high absorption coefficient. In recent years, colloidal AgBiS_2_ NCs have typically been synthesized via the hot injection method using hexamethyldisilathiane (TMS) as the sulfur source. However, the cost of TMS is one of the biggest obstacles to large–scale synthesis of colloidal AgBiS_2_ NCs. Herein, we synthesized colloidal AgBiS_2_ NCs using oleylamine@sulfur (OLA–S) solution as the sulfur source instead of TMS and optimized the synthesis conditions of colloidal AgBiS_2_ NCs. By controlling the reaction injection temperature and the dosage of OLA–S, colloidal AgBiS_2_ NCs with adjustable size can be synthesized. Compared with TMS–based colloidal AgBiS_2_ NCs, the colloidal AgBiS_2_ NCs based on OLA–S has good crystallinity and fewer defects.

## 1. Introduction

Colloidal AgBiS_2_ nanocrystal (NC), a member of the I–V–VI_2_ ternary semiconductor materials [1,2,3,4,5,6,7], is a promising eco–friendly material for solar cells owing to colloidal AgBiS_2_ NC having a wide absorption spectrum in the visible to near–infrared region (300–1600 nm) [8,9,10], high absorption coefficient (~10^5^ cm^−1^) [1,11,12,13], and good air stability [8]. The hot injection method is widely used to prepare high quality NCs such as PbS [14], PbSe [15,16], and halide perovskite NCs [17,18,19,20,21,22]. In 2016, Bernechea et al. synthesized colloidal AgBiS_2_ NCs by the hot injection method using hexamethyldisilathiane (TMS) as the sulfur source and first reported colloidal AgBiS_2_ NC–based solar cells with a certified power conversion efficiency (PCE) of 6.3% [1]. Subsequently, colloidal AgBiS_2_ NC–based solar cells have gradually become one of the mainstays of new generation solar cells. Subsequently, Hu et al. synthesized colloidal AgBiS_2_ NCs using an improved amine–based synthesis route, improving the PCE of colloidal AgBiS_2_ NC–based solar cells by 30% using colloidal AgBiS_2_ NCs synthesized using a modified route [23]. Oh et al. improved the size distribution of colloidal AgBiS_2_ NCs by an optimized synthetic route, resulting in enhanced photovoltaic performance [24]. Akgul et al. synthesized colloidal AgBiS_2_ NCs at room temperature using an improved sulfur amine–based synthesis route, reducing the production price by 60% and achieving a PCE of 5.5% [25]. These results demonstrate that the quality of colloidal AgBiS_2_ NCs plays an important role in the performance of solar cells. Therefore, it is of extreme importance to synthesize monodispersed colloidal AgBiS_2_ NCs in the quantum confinement regime and with a high phase purity [26]. Yet, it should be noted that although synthesis of colloidal AgBiS_2_ NCs has been developed by several researchers [1,27,28,29,30], it remains challenging to synthesize colloidal AgBiS_2_ NCs with a perfect crystal structure and complete elimination of defects [31,32,33,34,35,36,37,38]. Furthermore, using expensive and unstable TMS as the sulfur source in colloidal AgBiS_2_ NCs synthesis process results in high cost of the synthesis reaction, which is not conducive to large–scale preparation of colloidal AgBiS_2_ NC. It is urgent to explore a method to synthesize high quality colloidal AgBiS_2_ NC with low cost.

As a cheap sulfur source, oleylamine@sulfur (OLA–S) solution has been widely used to synthesize high quality CdS [39], ZnS [40], and PbS NCs [41,42]. Moreover, as a ligand stabilizer, oleylamine can passivate the surface defects of NCs and regulate their growth [23,43]. Recently, Nakazawa et al. successfully synthesized colloidal AgBiS_2_ NCs with OLA–S as sulfur source [44]. However, the effect of OLA–S on the growth and quality of colloidal AgBiS_2_ NCs has not been studied in detail. In this work, in order to improve the quality of colloidal AgBiS_2_ NCs and reduce the preparation cost, we synthesized colloidal AgBiS_2_ NCs by hot injection method and using OLA–S as the sulfur source to replace TMS, then investigated the effect of OLA–S on the growth of colloidal AgBiS_2_ NCs. By regulating the dosage of OLA–S, high quality colloidal AgBiS_2_ NCs with low defect density and uniform size were prepared. It was found that the size of colloidal AgBiS_2_ NCs could be controlled by changing the reaction injection temperature. Compared with TMS–based colloidal AgBiS_2_ NCs, the colloidal AgBiS_2_ NCs based on OLA–S have good crystallinity and fewer defects. In addition, the synthesis cost is significantly reduced.

## 2. Materials and Methods

Chemicals and materials. Bismuth (III) acetate (Bi(OAc)_3_, 99.9%, Aladdin, China), silver acetate (AgOAc, 99.5%, Aladdin, China), oleic acid (OA, 90%, Sigma–Aldrich, USA), hexamethyldisilathiane (TMS, synthesis grade, Sigma–Aldrich, Switzerland), 1-Octadecene (ODE, 90%, Aladdin, China), oleylamine (OLA, 80–90%, Aladdin, China), sulfur (S, 99.5%, Sinopharm, China), acetone (99.5%, Sinopharm, China), toluene (99.5%, Sinopharm, China), hexane(97.0%, Sinopharm, China).

Synthesis of TMS–based colloidal AgBiS_2_ NCs. The TMS–based colloidal AgBiS_2_ NCs were synthesized following a hot injection method reported previously in the literature [29].

Synthesis of OLA–S–based colloidal AgBiS_2_ NCs. The synthesis of OLA–S–based colloidal AgBiS_2_ NCs was carried out similarly to that of TMS–based colloidal AgBiS_2_ NCs. The OLA–S (1 M) solution was prepared by dissolving sulfur powder in OLA solution at room temperature. To investigate the influence of the reaction injection temperature (80 °C, 100 °C, 120 °C) on synthesis of OLA–S–based colloidal AgBiS_2_ NCs, the dosage of OLA–S solution (1 M) was kept at 3 mL. In contrast, to investigate the influence of the dosage of OLA–S solution (1 mL, 2 mL, 3 mL and 4 mL) on synthesis of OLA–S–based colloidal AgBiS_2_ NCs, the reaction injection temperature was kept at 100 °C. After rapidly injected the OLA–S solution into the cationic solution, heating was stopped while stirring was maintained until the reaction solution cooled to room temperature. The colloidal AgBiS_2_ NCs were collected using an acetone/toluene solvent system in air, and the obtained colloidal AgBiS_2_ NCs precipitate was then dried in a vacuum drying oven at room temperature. Finally, the colloidal AgBiS_2_ NCs were dispersed in hexane solution.

### Characterization

The phase identification of colloidal AgBiS_2_ NCs was carried out using powder X–ray diffraction (XRD, Bruker, D8 ADVANCED, Germany). The morphology and crystal structure of the prepared colloidal AgBiS_2_ NCs were characterized using high–resolution transmission electron microscopy (HR–TEM, Thermo Fisher Scientific, Talos F200x, USA). The TEM samples were prepared as follows: the obtained colloidal AgBiS_2_ NCs were dispersed in hexane with a concentration of 2 mg/mL, three drops of the mixture solution were dropped onto the TEM support film via pipette gun, and the TEM support films were fully dried in the vacuum drying oven before measurement. The acceleration voltage of electron beam was 200 KV during the TEM test. Elemental mapping of the colloidal AgBiS_2_ NCs was carried out using a TEM equipped with an energy dispersive spectrometer (EDS). UV–vis–NIR absorption spectra of the colloidal AgBiS_2_ NCs were recorded with a spectrophotometer (Shimadzu, UV–3600 PLUS, Japan). Fourier transform–infrared spectra (FT–IR) of colloidal AgBiS_2_ NCs were recorded using an infrared spectrometer (Bruker, INVENIO, Germany). The photoluminescent (PL) spectra of the colloidal AgBiS_2_ NCs were measured by using a fluorescence spectrophotometer (Zolix instruments Co., Ltd, FST1–MPL303–OP1, Beijing, China).

## 3. Results and Discussion

**Figure 1:**
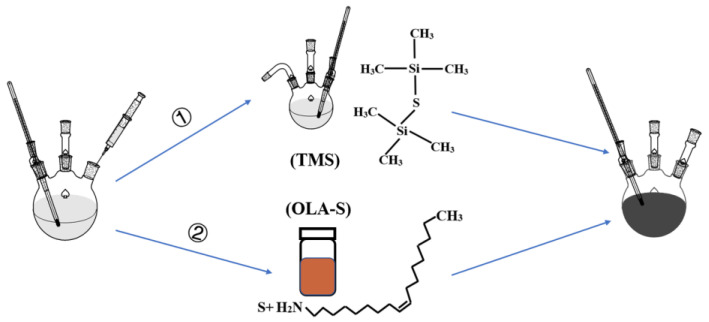
Synthetic route for colloidal AgBiS_2_ NCs using different sulfur sources (path 1: TMS, path 2: OLA–S).

**Figure 2:**
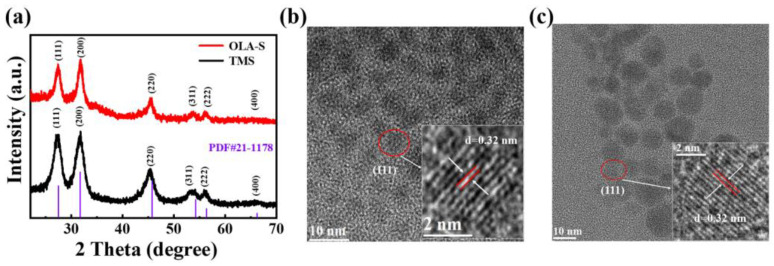
(**a**) XRD patterns of colloidal AgBiS_2_ NCs based on TMS and OLA–S; (**b**,**c**) HR–TEM images of TMS–based and OLA–S–based colloidal AgBiS_2_ NCs, respectively.

Following the schematic diagram of the synthesis of colloidal AgBiS_2_ NCs by hot injection method, which is shown in Figure 1, we adopted two methods to synthesize colloidal AgBiS_2_ NCs using TMS (path 1) and OLA–S (path 2) as the sulfur source. The variation of the crystal structure and phase of TMS–based and OLA–S–based colloidal AgBiS_2_ NCs were studied using X–ray diffraction (XRD). Figure 2a shows the XRD patterns of the TMS–based and OLA–S–based colloidal AgBiS_2_ NCs (dosage of OLA–S: 3 mL, injection temperature: 100 °C). For both samples, six XRD peaks are found at 13.75°, 15.85°, 22.89°, 27.12°, 28.20°, and 33.11°, which can be assigned to the facets (111), (200), (220), (311), (222), and (400) of AgBiS_2_ cubic rock salt structure (JCPDS No. 21–1178), respectively [8,12,37,45,46,47]. This indicates that colloidal AgBiS_2_ NCs can be successfully prepared by using OLA–S as the sulfur source. For NC materials, the broadening of XRD peaks can be roughly attributed to the size effect of NCs and lattice strain, which is closely related to defects in the NCs [48]. The lattice strains in TMS–based and OLA–S–based colloidal AgBiS_2_ NCs were calculated using the Williams–Hall plot method [49], with the results shown in Figure S1 and the corresponding Table S1. The obtained values of the lattice strains for TMS–based and OLA–S–based colloidal AgBiS_2_ NCs are 0.33 and 0.27, respectively. It can be seen that the lattice strain for TMS–based colloidal AgBiS_2_ NCs is larger than that of the OLA–S–based NCs, with the larger lattice strain leading to more defects in the NCs. Thus, the results demonstrate that OLA–S–based colloidal AgBiS_2_ NCs have lower defect density than TMS–based NCs. In other words, as a new sulfur source, OLA–S can be used to synthesize high quality colloidal AgBiS_2_ NCs.

Furthermore, in order to reveal the influence of the sulfur source on the morphology and size distribution of colloidal AgBiS_2_ NCs, transmission electron microscopy (TEM) measurement was carried out. Figure S2 shows that colloidal AgBiS_2_ NCs prepared using TMS and OLA–S have a uniform spherical shape. The average diameters of TMS–based and OLA–S–based colloidal AgBiS_2_ NCs are about 4.3 ± 1.6 nm (Figure S2a) and 7.8 ± 1.9 nm (Figure S2b), respectively. High–resolution TEM (HR–TEM) images of spherical colloidal AgBiS_2_ NCs based on TMS and OLA–S are shown in Figure 2b,c [50,51,52]. Lattice spacing of about 0.32 nm, corresponding to (111) crystal plane spacing of cubic rock salt structure AgBiS_2_, can be clearly seen, which is consistent with the XRD results above. Elemental mapping of TMS–based colloidal AgBiS_2_ NCs was carried out using a TEM equipped with EDS. Elemental mapping images are provided in Figure S3a–c, where the Ag, Bi, and S elements are distributed throughout all NCs. Moreover, the atomic percentages of Ag, Bi, and S elements are 41.3, 22.3, and 36.4, respectively. These results indicate that the surface of TMS–based colloidal AgBiS_2_ NCs should be silver–rich and sulfur–poor. Figure S3d–f shows the element mapping images of OLA–S–based colloidal AgBiS_2_ NCs. The atomic percentages of Ag, Bi, and S elements are 42.6, 12.3, and 45.1, respectively. This means that the surface of OLA–S–based colloidal AgBiS_2_ NCs is both silver–rich and sulfur–rich. Combined with the XRD result of the OLA–S–based colloidal AgBiS_2_ NCs, the signal of Ag_2_S is not observed in Figure 2a. Therefore, we speculate that the excess Ag and S are coating on the surface of colloidal AgBiS_2_ NCs rather than forming Ag_2_S impurities.

**Figure 3:**
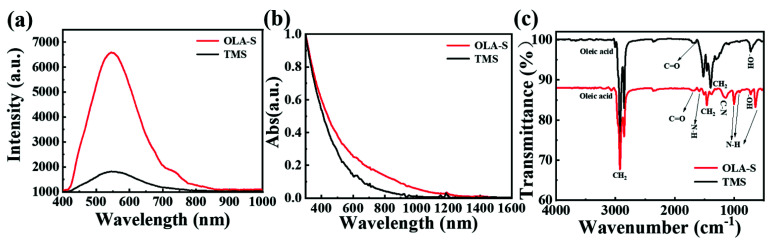
(**a**) PL spectra of TMS–based and OLA–S–based colloidal AgBiS_2_ NCs, (**b**) UV–vis–NIR absorption spectra of the colloidal AgBiS_2_ NCs in hexane, and (**c**) FT–IR spectra of colloidal AgBiS_2_ NCs.

To further reveal the effect of the sulfur source on the quality of the obtained colloidal AgBiS_2_ NCs, the photoluminescent (PL) spectra of the TMS–based and OLA–S–based colloidal AgBiS_2_ NCs solutions were measured at room temperature [53]. The PL spectra of colloidal AgBiS_2_ NCs based on TMS and OLA–S are shown in Figure 3a. We detected PL signals in the emission range of 400–1000 nm using a 330 nm excitation wavelength. It can be seen that the PL intensity of OLA–S–based colloidal AgBiS_2_ NCs is stronger than that of TMS–based colloidal AgBiS_2_ NCs under the same concentration. This indicates that there are fewer nonradiative charge recombination centers, which is closely related to lower defect density, in the OLA–S–based colloidal AgBiS_2_ NCs compared to the TMS–based colloidal AgBiS_2_ NCs.

The UV–vis–NIR absorption spectra of TMS–based and OLA–S–based colloidal AgBiS_2_ NCs are shown in Figure 3b. It can be seen that the absorption spectra of both of TMS–based and OLA–S–based colloidal AgBiS_2_ NCs do not exhibit any exciton absorption peak or sharp absorption edge in the range of 300–1600 nm, which is similar to the previously reported colloidal AgBiS_2_ NCs and other ternary semiconductor NCs such as colloidal CuInS_2_, AgInS_2_ and AgBiSe_2_ NCs [6,54,55,56]. The absorption spectrum of OLA–S–based colloidal AgBiS_2_ NCs extends to a larger IR region than the TMS–based colloidal AgBiS_2_ NCs, which may be owing to the size effect of larger grain size of OLA–S–based colloidal AgBiS_2_ NCs. This result is consistent with the result of the TEM images shown above.

FT–IR spectroscopy was employed to investigate the functional groups on the obtained colloidal AgBiS_2_ NCs [44,57]. Figure 3c shows the FT–IR spectra of colloidal AgBiS_2_ NCs. For TMS–based colloidal AgBiS_2_ NCs, the =C–H stretching peak at 3006 cm^−1^, C–H absorption stretching peaks at 2961, 2929, and 2854 cm^−1^, and C=O stretching mode at 1678 cm^−1^ are assigned to OA ligands on the surface of colloidal AgBiS_2_ NCs [55,58]. For OLA–S–based colloidal AgBiS_2_ NCs, the peaks at 903 cm^−1^, 721 cm^−1^, and 641 cm^−1^ are attributed to N–H vibrations of OLA. Additionally, a C–N stretching mode peak was observed at 1145 cm^−1^. The peak at 1678 cm^−1^ can be assigned to C=O stretching of OA ligands, indicating that the OLA–S–based colloidal AgBiS_2_ NCs are coated by OLA and OA ligands and that there are no amide–type ligands on the surface of the colloidal AgBiS_2_ NCs.

**Figure 4:**
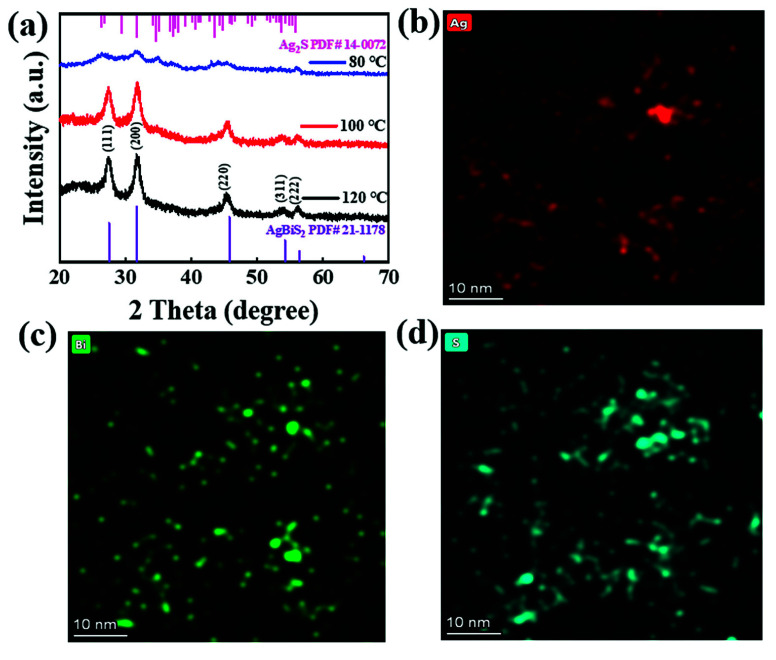
(**a**) XRD patterns of OLA–S–based colloidal AgBiS_2_ NCs synthesized at 80 °C, 100 °C, and 120 °C; (**b**–**d**) EDS elemental mapping (TEM) images of OLA–S–based colloidal AgBiS_2_ NCs prepared at 120 °C.

In order to reveal the influence of reaction temperature on the synthesis of OLA–S–based colloidal AgBiS_2_ NCs, a synthetic experiment was carried out at 80 °C, 100 °C, and 120 °C. The crystal structure and phase transition of colloidal AgBiS_2_ NCs based on OLA–S were investigated using XRD. Figure 4a shows the XRD patterns of OLA–S–based colloidal AgBiS_2_ NCs which were synthesized under different injection temperatures. The results show that colloidal AgBiS_2_ NCs can be successfully prepared at 100 °C and 120 °C. For sample prepared at 80 °C, the weak diffraction peak intensity indicates poor crystallinity of the obtained product and there are peaks belonging to monoclinic structure Ag_2_S (JCPDS No. 14–0072) at which impurities can be found. Therefore, pure colloidal AgBiS_2_ NCs cannot be synthesized at low temperature when using OLA–S as the sulfur source. The lattice strains of OLA–S–based colloidal AgBiS_2_ NCs obtained at 100 °C and 120 °C were calculated using the Williams–Hall plot from Figure S4, the corresponding results are shown in Table S2. The values of the lattice strains for OLA–S–based colloidal AgBiS_2_ NCs prepared at 100 °C and 120 °C are 0.27 and 0.38, respectively. It can be seen that the lattice strain of OLA–S–based colloidal AgBiS_2_ NCs produced at 120 °C is greater than the lattice strain OLA–S–based colloidal AgBiS_2_ NCs prepared at 100 °C, with larger lattice strain leading to more defects in the NCs. These results show that the defect density OLA–S–based colloidal AgBiS_2_ NCs prepared at 100 °C was lower than that of OLA–S–based NCs produced at 120 °C, meaning that 100 °C can be used to synthesize high quality colloidal AgBiS_2_ NC.

TEM measurement was used to reveal the influence of reaction temperature on the morphology and size distribution of OLA–S–based colloidal AgBiS_2_ NCs. Figure S5 shows that the colloidal AgBiS_2_ NCs prepared at 120 °C have a uniform spherical shape and that the average diameter of the obtained NCs is 12.1 ± 3.5 nm (Figure S5a). An HR–TEM image of colloidal AgBiS_2_ NCs prepared at 120 °C is shown in Figure S5b. The cubic rock salt structure of AgBiS_2_ with a lattice spacing of about 0.32 nm corresponding to the (111) crystal plane spacing can be seen, and is consistent with the XRD results. Figure 4b–d provides elemental diagram images of OLA–S–based colloidal AgBiS_2_ NCs synthesized at 120 °C. The atomic percentages of Ag, Bi, and S are 48.4, 17.1, and 34.6, respectively. These results show that the surface of the obtained colloidal AgBiS_2_ NCs should be silver–rich and sulfur–rich. Because the lattice strain of the OLA–S–based colloidal AgBiS_2_ NCs prepared at 120 °C is larger than that prepared at 100 °C, high reaction temperature may be detrimental to the preparation of high quality OLA–S–based colloidal AgBiS_2_ NCs.

Figure S6a shows the UV–vis–NIR absorption spectra of OLA–S–based colloidal AgBiS_2_ NCs prepared at 100 °C and 120 °C. The absorption spectrum of the sample synthesized at 120 °C extends to more IR regions than that of the NCs prepared at 100 °C, which is mainly caused by the size effect of the colloidal AgBiS_2_ NCs. To further reveal the effect of reaction injection temperature on the quality of the obtained colloidal AgBiS_2_ NCs, the PL spectra of OLA–S–based colloidal AgBiS_2_ NCs prepared at 100 °C and 120 °C were measured using 330 nm excitation wavelength at room temperature. The PL intensity of OLA–S–based colloidal AgBiS_2_ NCs synthesized at 100 °C is stronger than that of NCs synthesized at 120 °C (Figure S6b). This reflects that the OLA–S–based colloidal AgBiS_2_ NCs prepared at 100 °C has fewer defects than the colloidal AgBiS_2_ NCs synthesized at 120 °C.

**Figure 5:**
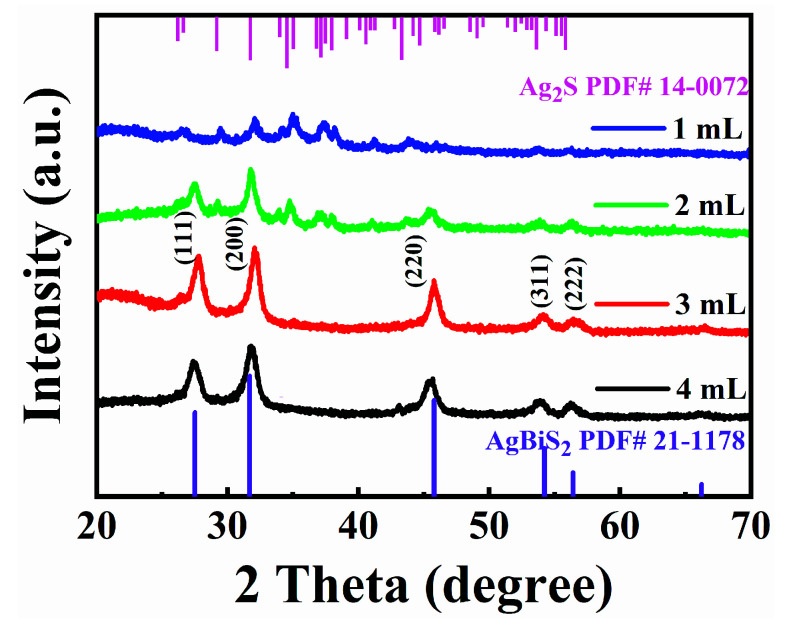
XRD patterns of colloidal OLA–S–based AgBiS_2_ NCs using 1 mL, 2 mL, 3 mL, and 4 mL.

In addition, the influence of OLA–S dosage in the reaction when synthesizing colloidal AgBiS_2_ NCs was investigated. We carried out the synthesis of colloidal AgBiS_2_ NCs using different amount of OLA–S (1 M). The crystallinity and phase purity of OLA–S–based colloidal AgBiS_2_ NCs synthesized under different conditions were examined by XRD. (Figure 5). It can be seen that only Ag_2_S was produced in the reaction when using 1 mL OLA–S, while both Ag_2_S and AgBiS_2_ were formed when the dosage of OLA–S was increased to 2 mL. Thus, pure phase colloidal AgBiS_2_ NCs can be successfully prepared using 3 mL and 4 mL OLA–S. Based on these results, we speculate that the reaction process of OLA–S–based colloidal AgBiS_2_ NC synthesis should be that: H_2_S being released in situ from OLA–S after OLA-S was injected into high temperature cationic solution [59], and then reacting with Ag^+^ to form Ag_2_S, subsequently, the produced Ag_2_S reacts with Bi^3+^ to form AgBiS_2_ NCs. The reaction equations are as follows:

(1) $$2{\mathrm{Ag}}^{+}+H_{2}S\;\to {\mathrm{Ag}}_{2}S+2H^{+},$$

(2) $$2{\mathrm{Ag}}_{2}S+{\mathrm{Bi}}^{3+}\to {\mathrm{AgBiS}}_{2}+3{\mathrm{Ag}}^{+}$$

The lattice strain values calculated for the colloidal AgBiS_2_ NCs prepared by using 3 mL and 4 mL OLA–S are 0.27 and 0.40, respectively, as shown in Figure S7 and Table S3. The smaller lattice strain in colloidal AgBiS_2_ NCs prepared using 3 mL OLA–S indicates a lower defect density in the NCs. To clarify the effect of the dosage of OLA–S on the morphology and size distribution of colloidal AgBiS_2_ NCs, we carried out HR–TEM measurements of the obtained AgBiS_2_ NCs. Figure S8a shows that colloidal AgBiS_2_ NCs prepared using 4 mL OLA–S have a uniform spherical shape and the average diameters of NCs are about 8.3 ± 2.7 nm, which is a little larger than that based on 3 mL OLA–S (7.8 ± 1.9 nm; see Figure S2b). Figure S6c shows the UV–vis–NIR absorption spectra of 3 mL and 4 mL OLA–S–based colloidal AgBiS_2_ NCs. The absorption spectrum of the 4 mL OLA–S–based colloidal AgBiS_2_ NCs extends to more IR regions than that of the 3 mL OLA–S–based colloidal AgBiS_2_ NCs, which is mainly caused by the size effect of the colloidal AgBiS_2_ NCs.

Finally, we calculated the cost and yield of the synthesis of colloidal AgBiS_2_ NCs using TMS and OLA–S as sulfur sources. Compared with TMS, the cost of synthesis using OLA–S was reduced about 57% (see Table S4 for details). We found that the yield of colloidal AgBiS_2_ NCs based on TMS and OLA–S were 86.9% and 90.5%, respectively. This indicates that OLA–S can replace TMS for synthesizing colloidal AgBiS_2_ NCs as a low–cost sulfur source.

## 4. Conclusions

In summary, we optimized the synthesis conditions of colloidal AgBiS_2_ NCs using OLA–S as the sulfur source. Colloidal AgBiS_2_ NCs with high quality and pure phase can be synthesized by rationally optimizing the dosage and reaction injection temperature of OLA–S. Compared with TMS as the sulfur source, OLA–S–based colloidal AgBiS_2_ NCs have lower defect density and a wider absorption spectrum. More importantly, the cost of synthesis is greatly reduced, as the yield of colloidal AgBiS_2_ NCs is increased when using OLA–S to replace the more expensive TMS. In addition, the pungent odor and air sensitivity of TMS can be avoided. As a low–cost sulfur source, OLA–S has the potential to for use in synthesizing other sulfide NCs in the future.

## Data Availability

The data that support the findings of this study are available from the corresponding author upon reasonable request.

## Supplementary Materials

The supporting information can be downloaded at: https://www.mdpi.com/article/10.3390/nano12213742/s1. Figure S1. The William–Hall plots for (a) TMS–based and (b) OLA–S–based colloidal AgBiS_2_ NCs. Figure S2. TEM images of (a) TMS–based and (b) OLA–S–based colloidal AgBiS_2_ NCs. Insets are the statistical histograms of the NCs size distribution. SAED images of (c) TMS–based and (d) OLA–S–based colloidal AgBiS_2_ NCs. Figure S3. EDS–Mapping images of (a), (b) and (c)TMS–based. EDS–Mapping images of (d), (e) and (f) OLA–S–based colloidal AgBiS_2_ NCs. Figure S4. The William–Hall plots for (a) and (b) OLA–S–based colloidal AgBiS_2_ NCs prepare at 100 °C and 120 °C. Figure S5. TEM images of (a) OLA–S–based colloidal AgBiS_2_ NCs prepare at 120 °C. Insets are the statistical histograms of the NCs size distribution. (b) HR–TEM images of OLA–S–based colloidal AgBiS_2_ NCs prepare at 120 °C. (c) SAED image of OLA–S–based colloidal AgBiS_2_ NCs prepare at 120 °C. Figure S6. (a) UV–vis–NIR absorption spectra of the colloidal AgBiS_2_ NCs in hexane. (b) PL spectrum and fitting curves of OLA–S–based colloidal AgBiS_2_ NCs prepare at 100 °C and 120 °C. (b) UV–vis–NIR absorption spectra of the 3 mL and 4 mL colloidal AgBiS_2_ NCs in hexane. Figure S7. The William–Hall plots for (a) 3 mL and (b) 4 mL OLA–S–based colloidal AgBiS_2_ NCs. Figure S8. TEM images of (a) 4 mL OLA–S–based colloidal AgBiS_2_ NCs. Insets are the statistical histograms of the NCs size distribution. (b) HR-TEM images of 4 mL OLA–S–based colloidal AgBiS_2_ NCs. (c) SAED image of 4 mL OLA–S–based colloidal AgBiS_2_ NCs. Table S1. Half–peak widths and lattice strains of OLA–S–based and TMS–based colloidal AgBiS_2_ NCs. Table S2. Half–peak widths and lattice strains of OLA–S–based colloidal AgBiS_2_ NCs prepare at 100 °C and 120 °C. Table S3. Half–peak widths and lattice strains of 3 mL and 4 mL OLA–S–based colloidal AgBiS_2_ NCs. Table S4. Raw material usage, price and yield of AgBiS_2_ NCs based on TMS and OLA–S. Reference [60] is cited in the Supplementary Materials.
