# Supplementary material for: Optimizing the Synthetic Conditions of “Green” Colloidal AgBiS2 Nanocrystals Using a Low-Cost Sulfur Source
Source: Nanomaterials (Basel). 2022 Oct 25;12(21):3742. doi: 10.3390/nano12213742 (PMC9654632; doi:10.3390/nano12213742)
Supplement: Supplementary file 1 [file nanomaterials-12-03742-s001.zip › nanomaterials-1959998-supplementary.pdf]

Supporting Information

Optimizing the Synthetic Conditions of "Green" Colloidal $AgBiS_2$ Nanocrystals Using Low-Cost Sulfur Source

Qiao Li [1], Xiaosong Zheng [1], Xiaoyu Shen [1], Shuai Ding [1], Hongjian Feng [1,*], Guohua Wu [2,*], Yaohong Zhang [1,3*]

[1] School of Physics, Northwest University, Xi'an 710127, China

[2] Qingdao Innovation and Development Base of Harbin Engineering University, Harbin Engineering University, Heilongjiang 150001, China

[3] Shaanxi Key Laboratory for Carbon Neutral Technology, Xi'an 710127, China

*Corresponding authors:

Yaohong Zhang: yhzhang@nwu.edu.cn

Hong-Jian Feng: hjfeng@nwu.edu.cn

Guohua Wu: ghwu@hrbeu.edu.cn

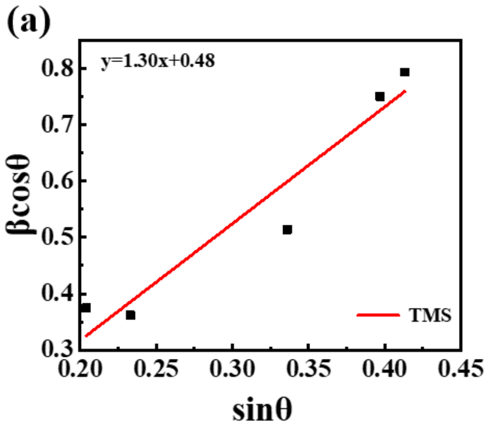

Figure S1. The William-Hall plots for (a) TMS-based and (b) OLA-S-based colloidal $AgBiS_2$ NCs.

The relationship between full width at half maximum of XRD peak (β) and lattice strain (ε) follows the Williamson-Hall equation: [1,2]

$$\beta \cos\theta = 4\varepsilon \sin\theta + \frac{K\lambda}{D_{size}} \qquad \text{eq. (1)}$$

where θ is the Bragg angle in radian, K is the sharp factor (K=0.94), λ is the wavelength of X-ray (CuK_β) in nanometers (λ=0.14 nm), and D_{size} is the average NC size in nanometers, respectively. The lattice strain, ε, can be evaluated from the slope of the linear fitting of the data when βcosθ is plotted on the y axis and sinθ is plotted on the x axis. The fitting results are shown in Table S1.

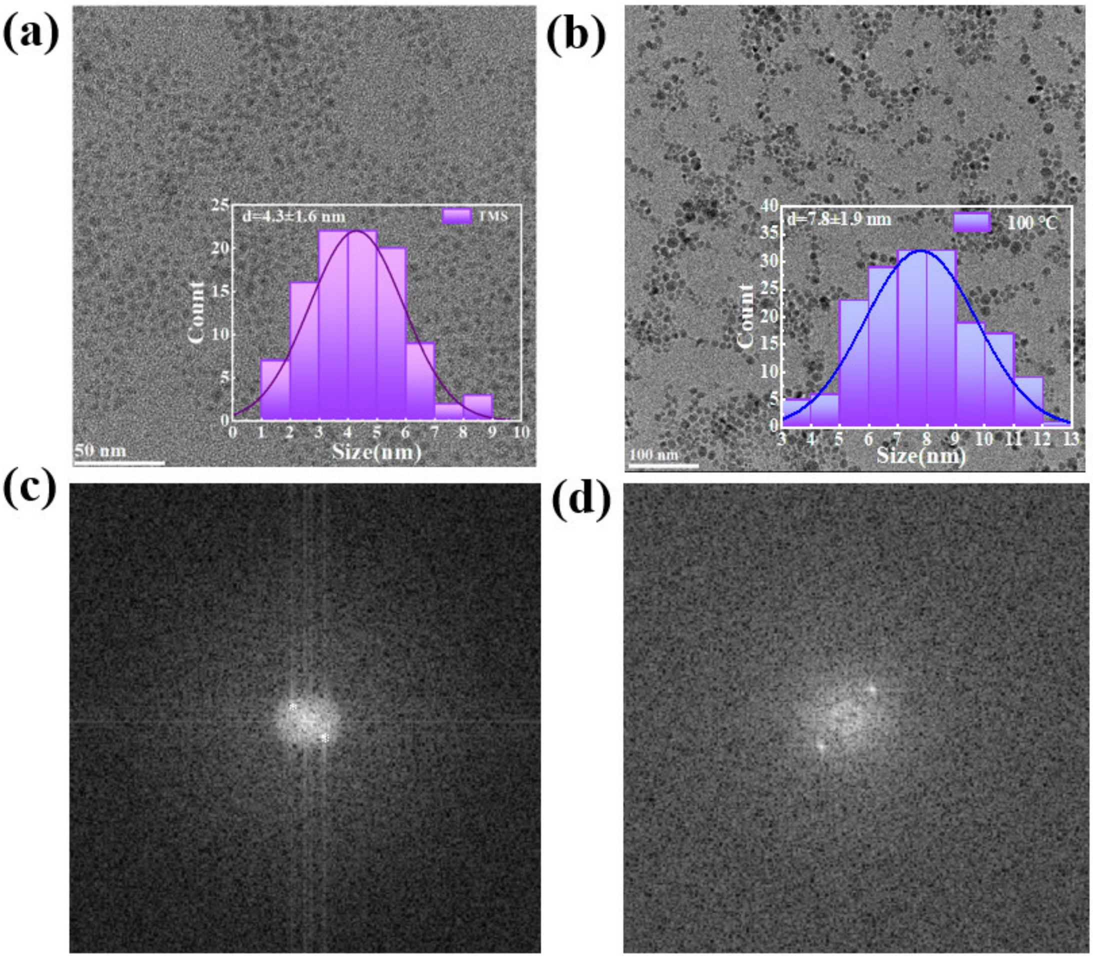

Figure S2. TEM images of (a) TMS-based and (b) OLA-S-based colloidal $AgBiS_2$ NCs. Insets are the statistical histograms of the NCs size distribution. SAED images of (c) TMS-based and (d) OLA-S-based colloidal $AgBiS_2$ NCs.

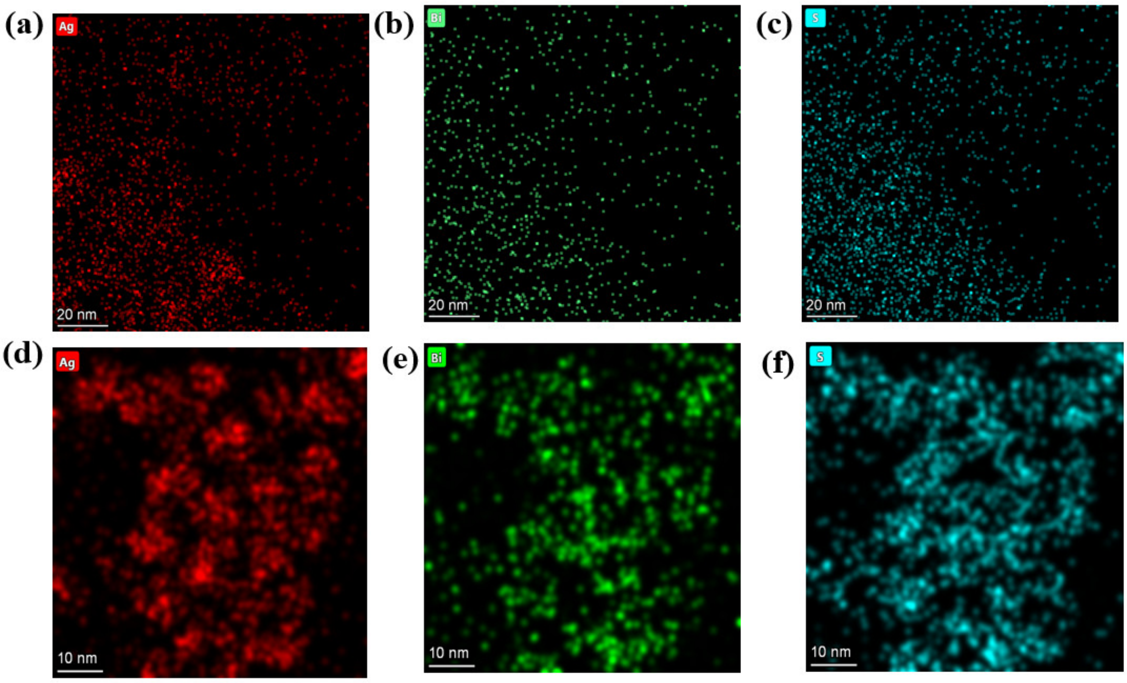

Figure S3. EDS-Mapping images of (a) Ag, (b) Bi and (c) S of TMS-based. EDS-Mapping images of (d) Ag, (e) Bi and (f) S of OLA-S-based colloidal $AgBiS_2$ NCs.

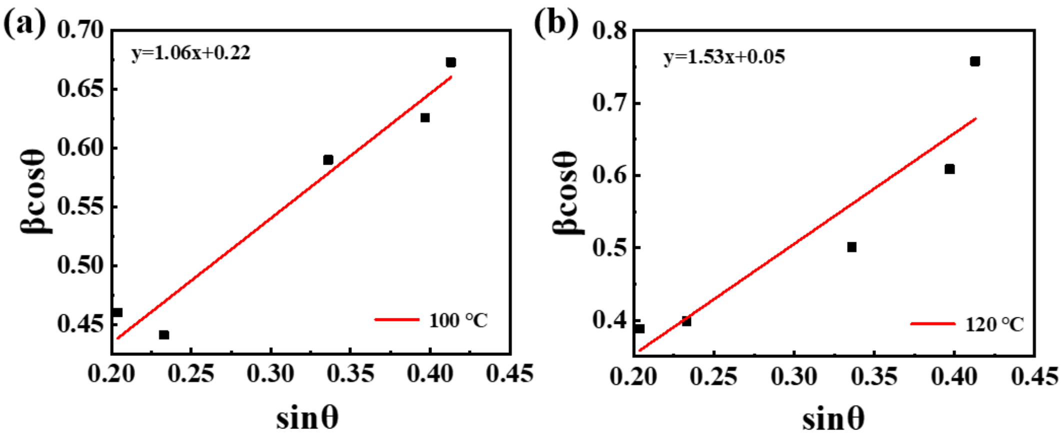

Figure S4. The William-Hall plots for OLA-S-based colloidal $AgBiS_2$ NCs prepare at (a) 100°C and (b) 120°C.

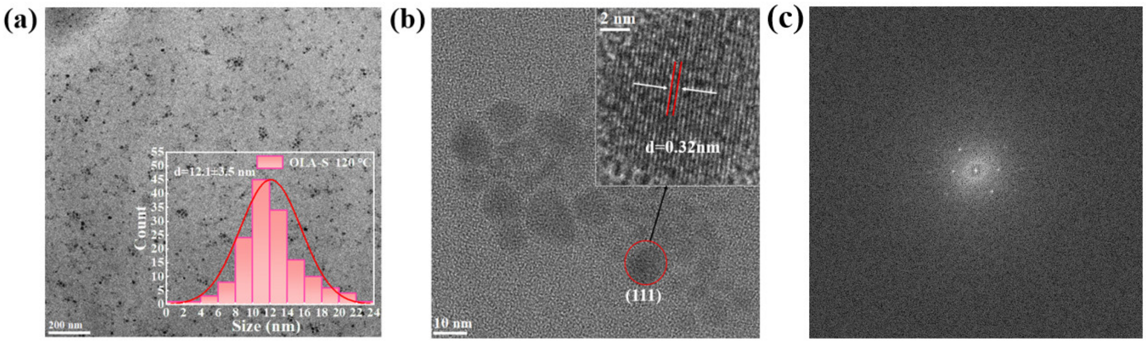

Figure S5. TEM images of (a) OLA-S-based colloidal $AgBiS_2$ NCs prepare at 120°C. Insets are the statistical histograms of the NCs size distribution. (b) HR-TEM images of OLA-S-based colloidal $AgBiS_2$ NCs prepare at 120°C. (c) SAED image of OLA-S-based colloidal $AgBiS_2$ NCs prepare at 120°C.

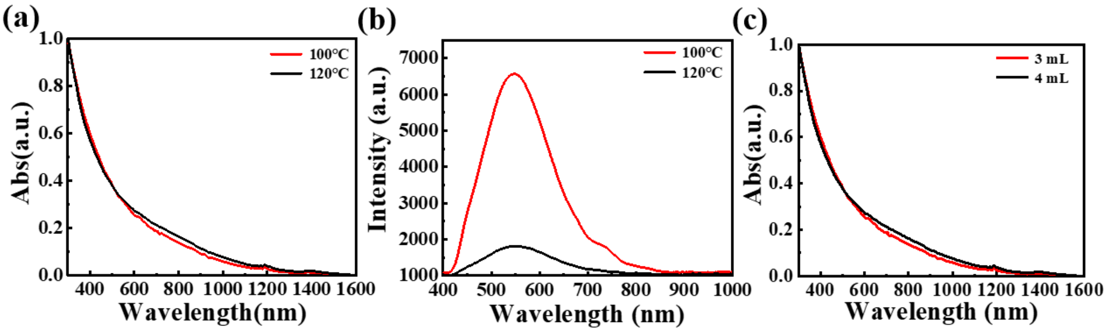

Figure S6. (a) UV−vis−NIR absorption spectra of the colloidal $AgBiS_2$ NCs in hexane. (b) PL spectrum and fitting curves of OLA-S-based colloidal $AgBiS_2$ NCs prepare at 100°C and 120°C. (c) UV−vis−NIR absorption spectra of the 3 mL and 4 mL colloidal $AgBiS_2$ NCs in hexane.

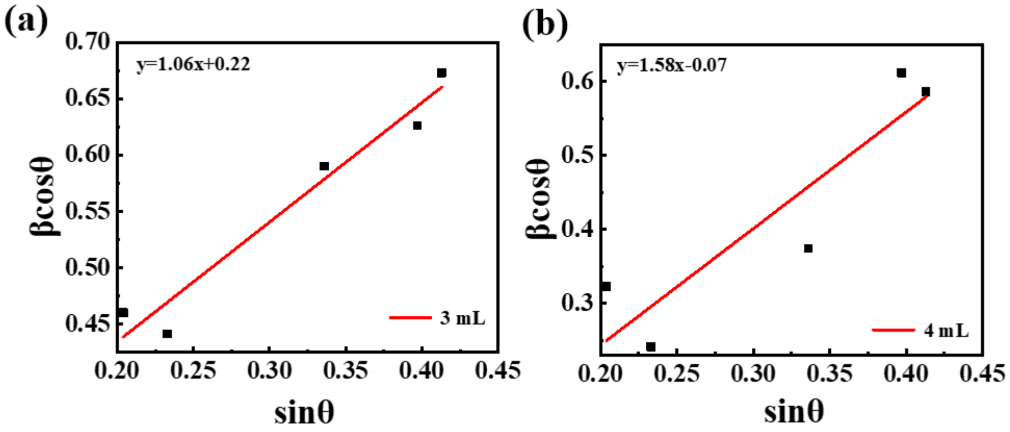

Figure S7. The William-Hall plots for (a) 3 mL and (b) 4 mL OLA-S-based colloidal $AgBiS_2$ NCs.

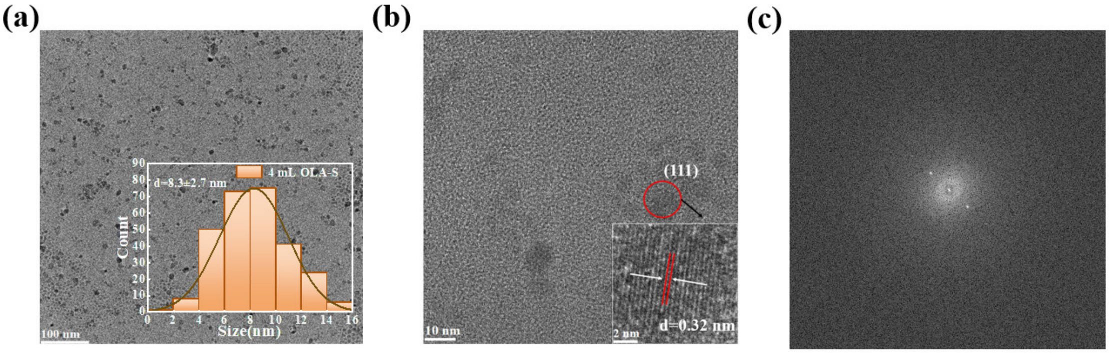

Figure S8. TEM images of (a) 4 mL OLA-S-based colloidal $AgBiS_2$ NCs. Insets are the statistical histograms of the NCs size distribution. (b) HR-TEM images of 4 mL OLA-S-based colloidal $AgBiS_2$ NCs. (c) SAED image of 4 mL OLA-S-based colloidal $AgBiS_2$ NCs.

Table S1. Half-peak widths and lattice strains of OLA-S-based and TMS-based colloidal $AgBiS_2$ NCs.

Sulfur source	Crystal plane	Half-peak wide(nm)	Lattice strain
OLA-S	(111)	3.51	0.27
	(200)	2.95	
	(220)	2.73	
	(311)	3.45	
	(222)	2.53	
TMS	(111)	2.81	0.33
	(200)	2.50	
	(220)	4.06	
	(311)	4.34	
	(222)	2.66	

Table S2. Half-peak widths and lattice strains of OLA-S-based colloidal $AgBiS_2$ NCs prepare at 100°C and 120°C.

Temperature (°C)	Crystal plane	Half-peak wide(nm)	Lattice strain
100°C	(111)	3.51	0.27
	(200)	2.95	
	(220)	2.73	
	(311)	3.44	
	(222)	2.53	
120°C	(111)	2.96	0.38
	(200)	2.66	
	(220)	2.32	
	(311)	2.38	
	(222)	2.29	

Table S3. Half-peak widths and lattice strains of 3 mL and 4 mL OLA-S-based colloidal $AgBiS_2$ NCs.

Temperature (°C)	Crystal plane	Half-peak wide(nm)	Lattice strain
3 mL	(111)	3.51	0.27
	(200)	2.95	
	(220)	2.73	
	(311)	3.44	
	(222)	2.53	
4 mL	(111)	2.46	0.40
	(200)	1.60	
	(220)	1.73	
	(311)	2.39	
	(222)	2.20	

Table S4. Raw material usage, price and yield of $AgBiS_2$ NCs based on TMS and OLA-S.

Sulfur Source	Raw Material	Synthetic Usage	Price(yuan)	Total(yuan)	Yield(%)
TMS	$Bi(OAc)_3$	386 mg	24.5	80.4	(265 mg) 86.9
	AgOAc	134 mg	3.0		
	OA	5.4 mL	4.3		
	ODE	15 mL	2.6		
	TMS	215 μL	46.0		
OLA-S	$Bi(OAc)_3$	386 mg	24.5	34.8	(276 mg) 90.5
	AgOAc	134 mg	3.0		
	OA	5.4 mL	4.3		
	ODE	10 mL	1.8		
	OLA	3 mL	1.2		
	S	96 mg	0.003		

Reference

1. Williamson, G. K.; Hall, W. H. X-ray line broadening from filed aluminium and wolfram L'elargissement des raies de rayons x obtenues des limailles d'aluminium et de tungsten Die verbreiterung der roentgeninterferenzlinien von aluminium- und wolframspaenen, *Acta Metallurgica* **1953**, *1*, 22-31.

2. Nishimura, K.; Hirotani, D.; Kamarudin, M. A.; Shen, Q.; Toyoda, T.; Iikubo, S.; Minemoto, T.; Yoshino, K.; Hayase, S. Relationship between lattice strain and efficiency for Sn-perovskite solar cells, *ACS Appl. Mater. Interfaces* **2019**, *11*, 31105-31110.
